# Encapsulation of C–N-decorated metal sub-nanoclusters/single atoms into a metal–organic framework for highly efficient catalysis†
†Electronic supplementary information (ESI) available. See DOI: 10.1039/c8sc03549k


**DOI:** 10.1039/c8sc03549k

**Published:** 2018-09-26

**Authors:** Xuan Qiu, Jianmin Chen, Xinwei Zou, Ruiqi Fang, Liyu Chen, Zhijie Chen, Kui Shen, Yingwei Li

**Affiliations:** a State Key Laboratory of Pulp and Paper Engineering , School of Chemistry and Chemical Engineering , South China University of Technology , Guangzhou 510640 , China . Email: cekshen@scut.edu.cn ; Email: liyw@scut.edu.cn

## Abstract

Fabrication and modification of few-atom metal clusters and even single atoms in the pores of porous materials for catalysis are highly desired from an atom-efficiency aspect but remain a great challenge. Herein, we propose a facile and efficient strategy for the encapsulation of C–N-decorated Pd sub-nanoclusters (MSNCs)/single atoms (SAs) into MOFs by the confined thermolysis of Pd-based metal–organic polyhedra (MOPs) in MOF pores. The obtained hybrids contained both Pd MSNCs (∼0.8 nm) and Pd SAs, which were stabilized by the *in situ* formed C–N fragments and the confinement effect of MOF pores. Benefiting from the highly exposed Pd atoms and synergistic effect between Pd and C–N fragments, these catalysts exhibited extremely high catalytic activity and stability in various important chemical processes, making them comparable to the most active Pd-based catalysts reported in the literature even under milder reaction conditions. Considering the high tunability of MOPs, this proposed strategy might provide a new toolbox for enriching the family of decorated MSNC/SA catalysts.

## Abstract 2

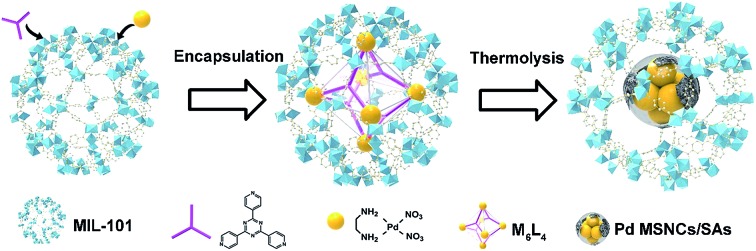
A facile strategy was designed for the encapsulation of C–N-decorated Pd sub-nanoclusters/single atoms into MOF pores by the confined thermolysis of metal–organic polyhedra (MOPs). The obtained hybrids exhibited excellent catalytic performance in various important chemical processes.

## Introduction

Owing to sustainable development in energy and environment areas, metal sub-nanoclusters (MSNCs) have attracted extensive attention due to their fascinating catalytic properties stemming from their high atom efficiencies.^1^,^2^ In particular, isolated single atoms (SAs) as an exceptional case feature atomically dispersed metal atoms and maximum atom utilization, which have resulted in superior catalytic performance in a number of reactions.^3^ Moreover, it has also been demonstrated that the catalytic performance of metal sub-nanoclusters (MSNCs) or/and even single atoms (SAs) could be further improved significantly upon functionalization (*e.g.*, C–N decoration) because such modifications are believed to be able to affect the electron distribution, size and stability of the catalysts.^4^ However, the traditional synthetic approaches for C–N decorated MSNCs/SAs always caused serious aggregation in high-temperature pyrolysis processes due to the ultra-high surface energy of the MSNCs/SAs.^5^ As is known, the strong interaction or/and confinement effect between the MSNCs/SAs and supports can well stabilize these MSNCs/SAs and prevent them from aggregating during both materials synthesis and catalytic reactions. Thus, exploring effective strategies to immobilize decorated MSNCs/SAs on robust supports for advanced catalysis applications is highly desirable but remains extremely challenging.

Metal–organic polyhedra (MOPs) are a new class of discrete coordination complexes built from self-assembly of inorganic metal ions and organic ligands.^6^ Owing to their well-defined structure and excellent symmetry, MOPs have demonstrated great potential for application in a variety of fields such as catalysis and molecular sensing.^7^ However, their practical applications are currently restricted due to instability of the MOP structures, which tend to collapse under harsh conditions such as high temperatures.^8^ Considering their ordered structures and isolated metal ions, we proposed that it could be feasible to employ MOPs as pyrolysis precursors for the fabrication of uniform functional composites such as C–N-decorated MSNCs/SAs by low-temperature thermolysis. During the pyrolysis procedure, the *in situ* formed C–N fragments derived from the organic ligands are suspected to play an important role in preventing serious aggregation of metal ions. Nevertheless, as far as we know, there is no report on employing MOPs as sacrificial precursors for the preparation of C–N-decorated MSNCs/SAs.

Herein, we report the fabrication of highly dispersed C–N-decorated MSNCs/SAs, which are encapsulated and stabilized in the pores of a metal–organic framework (MOF) by employing MOPs as a sacrificial template. MOFs are a newly developed class of porous materials featuring a uniform structure and well-defined porosity.^9^ These properties make MOFs one of the most fantastic hosts for the encapsulation of various guests.^10^ Moreover, it has also been demonstrated that the confinement could significantly enhance the stabilities and properties of the encapsulated guests.^11^ Our developed strategy employed MOPs as a sacrificial template, which was firstly encapsulated into the MOF pores and then pyrolyzed. By taking advantage of the thermal instability difference between the MOP and the MOF, the MOP guest collapsed to form C–N-decorated MSNCs/SAs while the MOF host preserved its crystalline framework. The residual C–N fragments derived from the organic ligands of the MOP were supposed to act as *in situ* formed stabilizers to prevent the metal from aggregating, achieving highly dispersed C–N-decorated MSNCs/SAs, which were further stabilized by the confinement effect offered by the MOF cages. This “benign by design” strategy may provide new insights into preparation of highly active and stable MSNC/SA systems.

## Results and discussion

**Scheme 1:**
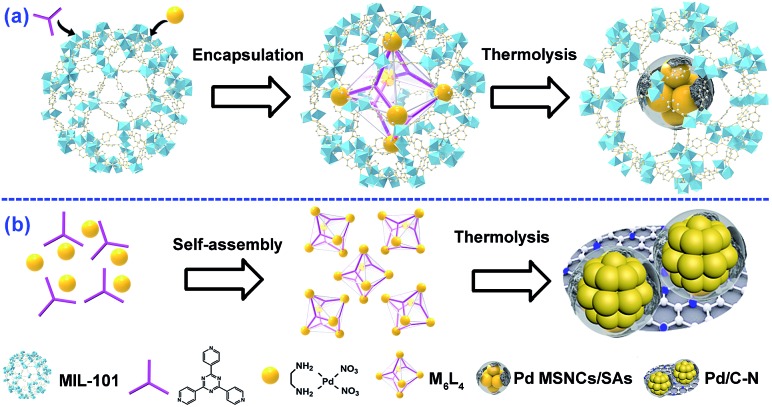
Schematic illustration of (a) PCN⊂M and (b) Pd/C–N synthesis.

As a proof of principle, MIL-101(Cr) was employed as the host and M_6_L_4_ (**M** stands for (en)Pd(NO_3_)_2_; **L** stands for 1,3,5-tris(4-pyridyl)-2,4,6-triazine, also named TPT) was used as a model MOP. As a typical MOF, MIL-101(Cr) possesses two types of mesopores with internal free diameters of *ca.* 29 Å and 34 Å.^12^ M_6_L_4_ is a hollow octahedral self-assembly with six vertices occupied by six cationic **M** and eight triangular faces alternately occupied by four triangular ligands **L**.^13^ Typically, this strategy involves two steps (Scheme 1a): (1) encapsulating M_6_L_4_ into MIL-101 pores by a hydrophilicity-directed approach (HDA) to obtain the M_6_L_4_⊂MIL-101 hybrids,8*a* and (2) pyrolyzing the hybrids under a H_2_ flow. The obtained materials were denoted as Pd/C–N⊂MIL-101 (or PCN⊂M for simplicity).

Thermogravimetric analysis (TGA) was first carried out to measure the thermal stability of M_6_L_4_ and MIL-101 in order to obtain the optimized pyrolysis temperature. As shown in Fig. S1,† the decomposition temperature for M_6_L_4_ and MIL-101 was 230 °C and 350 °C respectively. H_2_-TPR measurement was also performed to investigate the redox properties of M_6_L_4_ and M_6_L_4_⊂MIL-101. As shown in Fig. S1a,† M_6_L_4_ showed a main reduction peak at 215 °C, which can be ascribed to the reduction of Pd^2+^ to Pd^0^. Similarly, a strong reduction peak at *ca.* 250 °C was also detected for M_6_L_4_⊂MIL-101 due to the reduction of Pd^2+^ in its structure, indicating that Pd^0^ could be successfully prepared by reduction at 250 °C in a H_2_ atmosphere. Thus, we chose 250 °C as the optimal reduction temperature to prepare various PCN⊂M. In addition, M_6_L_4_ was also pyrolyzed at 250 °C under H_2_ flow (Scheme 1b) for comparison. The residual composite was named Pd/C–N due to the presence of Pd, C, and N elements, as indicated by the elemental analysis and AAS (Table S1†). The powder X-ray diffraction (PXRD) patterns of the Pd/C–N (Fig. S2†) showed eight diffraction peaks, demonstrating the characteristics of metallic Pd (JCPDS no. 46-1043). Representative transmission electron microscopy (TEM) images (Fig. S3a†) indicated that the Pd particles in Pd/C–N have an irregular morphology (10–30 nm) with some C–N fragments wrapped around them. High-angle annular dark-field scanning transmission electron microscopy (HAADF-STEM) and the corresponding EDX elemental mapping (Fig. S3b–e†) revealed a uniform distribution of C and N in Pd-enriched NPs, which was further confirmed by the elemental line-scanning spectra (Fig. S3f†). These results clearly indicated that after thermolysis in H_2_, the Pd centers in M_6_L_4_ aggregated and reduced to Pd particles while the organic components collapsed to C–N fragment layers.

The XPS and Fourier transform infrared spectroscopy (FT-IR) spectra of the M_6_L_4_⊂MIL-101 (Fig. S4†) were similar to those reported, indicating the successful encapsulation of M_6_L_4_ into the MIL-101 pores.8*a* By treating M_6_L_4_⊂MIL-101 at 250 °C under a H_2_ flow, M_6_L_4_ decomposed while MIL-101 remained stable due to its higher thermo-stability (Fig. S1†), forming the PCN⊂M with 0.64 wt% Pd loading (denoted as 0.64PCN⊂M). Three other PCN⊂M with different Pd loadings were also prepared, which were named 0.33PCN⊂M, 0.51PCN⊂M and 0.82PCN⊂M based on their actual Pd content (Table S2†). The PXRD patterns of all PCN⊂M (Fig. S5†) hybrids were similar to that of MIL-101 and no characteristic peak of the Pd phase was observed, suggesting the well-preserved structure of the parent MIL-101 and the high dispersion of Pd in PCN⊂M.^12^,^14^ The transformation of M_6_L_4_ in M_6_L_4_⊂MIL-101 after pyrolysis was further confirmed by FT-IR. As presented in Fig. S6,† the spectrum of M_6_L_4_⊂MIL-101 exhibited distinct characteristic bonds of M_6_L_4_ with the 715 cm^–1^ peak assigned to NO_3_^–^ stretching in **M**, and the 673 cm^–1^ peak assigned to NH_2_ stretching in **L**.8*a* After heating in H_2_, these two peaks disappeared, indicating collapse of the M_6_L_4_ structure. However, the 1061 cm^–1^ peak assigned to C–N stretching remained even though its intensity decreased, which indicated that the C–N fraction was still preserved during the pyrolysis.

**Fig. 1:**
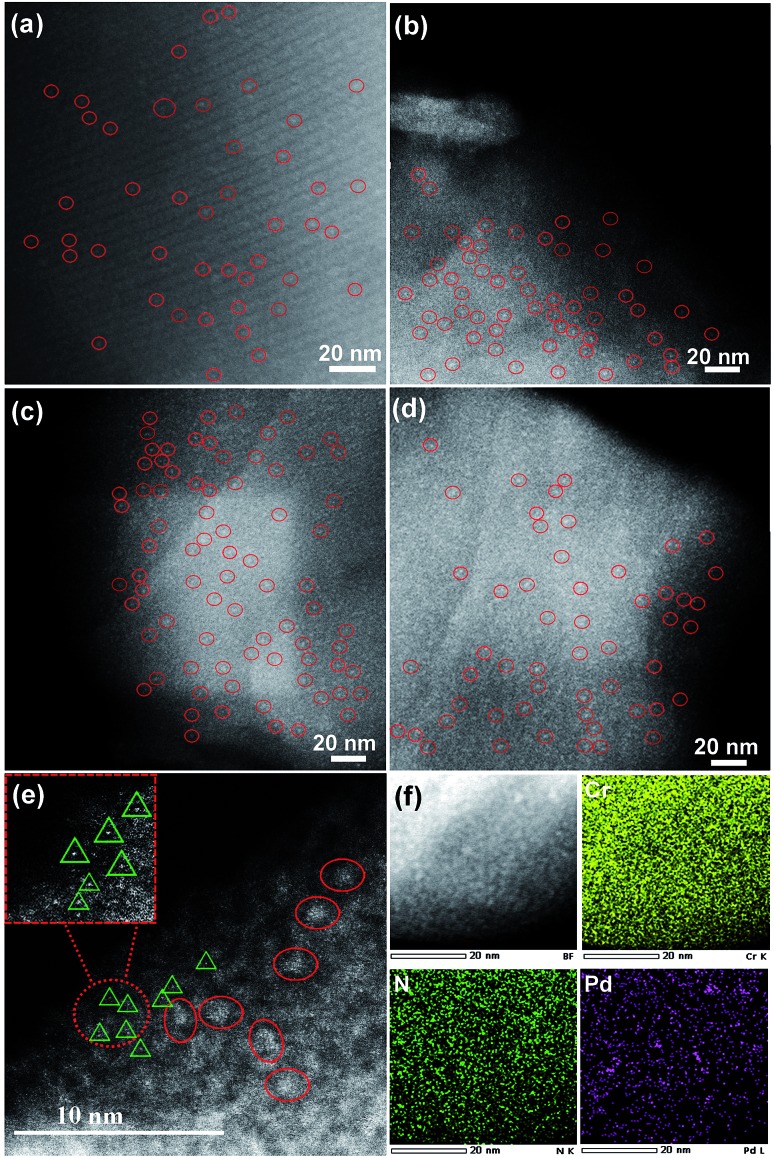
HAADF-STEM images and the corresponding size distribution of Pd sub-nanoclusters for ultrathin cuts from (a) 0.33PCN⊂M, (b) 0.51PCN⊂M, (c) 0.64PCN⊂M and (d) 0.82PCN⊂M. (e) Aberration-corrected HAADF-STEM image of 0.64PCN⊂M; the inset is the magnified image of the area enclosed by the dotted line. (f) The elemental mapping of 0.64PCN⊂M. The solid triangles and circles in the figures represent single atoms and sub-nanoclusters, respectively.

The structures of various samples were characterized in detail by HAADF-STEM. Clearly, M_6_L_4_⊂MIL-101 showed a smooth surface without clusters (Fig. S7†). After thermolysis, all PCN⊂M hybrids exhibited a uniform distribution of Pd sub-nanoclusters with ultrafine average particle sizes of *ca.* 0.8 nm (Fig. 1a–d and S8–S11†). Delightfully, further characterization of spherical aberration corrected electron microscopy revealed that 0.64PCN⊂M, as a representative, also showed plenty of bright spots besides bright clusters, which were assigned to the atomically dispersed Pd (Pd single atoms and Pd clusters are indicated by solid triangles and circles in Fig. 1e for clarity, respectively). The elemental mapping showed that Pd, N, and Cr were homogeneously dispersed (Fig. 1f). These results suggested that both Pd MSNCs and SAs were present and highly distributed in our system. In general, metal particles will aggregate seriously when the loading rises for support materials. However, we are delighted to mention that in our PCN⊂M system the main particle distribution was still located below the sub-nanoscale zone even when the Pd content was increased up to 0.82%.

**Fig. 2:**
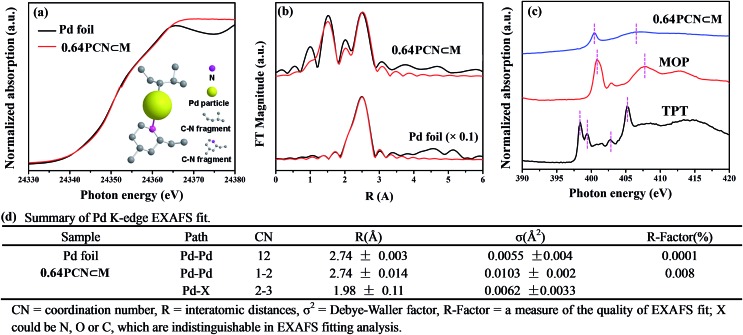
(a) Pd K-edge NEXAFS spectra of Pd foil and PCN⊂M; the inset is the schematic model of PCN⊂M. (b) Fourier transformed EXAFS of Pd foil and PCN⊂M (black) and their best fits (red). (c) N K-edge NEXAFS spectra of TPT, MOP, and PCN⊂M samples. (d) Summarized data of the Pd K-edge EXAFS fit.

The fine-structure information and chemical bonding environment were further studied by the extended X-ray absorption fine structure (EXAFS) and near-edge X-ray absorption fine structure (NEXAFS) (Fig. 2). In the Pd K-edge, the absorption threshold appearing between 24 330 and 24 365 eV, called the white line, corresponds to the 1s–4p electron transitions and is sensitive to variations of the electron occupancy in the valence band and ligand field environments of the absorber.3a,^15^ As shown in Fig. 2a, the white-line intensity of PCN⊂M was mostly similar to that of Pd foil except that the intensity was a little bit higher. This indicated that the Pd valence in PCN⊂M was similar to that in Pd foil but in a more oxidized form.^16^Fig. 2b shows the EXAFS Fourier transform (without phase correction) of Pd foil as a reference and PCN⊂M together with the results of the curve-fitting analysis. The coordination numbers (CNs) of Pd–Pd bonding (CN_Pd–Pd_ ≈ 1 to 2) in the PCN⊂M were as low as 1 to 2, and were significantly lower than that of bulk Pd foil (CN_Pd–Pd_ ≈ 12) (Fig. 2d), confirming the high dispersion of the Pd MSNCs/SAs encapsulated in the MIL-101.^17^ This result was also consistent with the HAADF-STEM results. PCN⊂M presented (Fig. 2d) two evident peaks at around 1.98 Å and 2.74 Å (after phase correction, the bond lengths *R* were larger than those without phase correction in Fig. 2b), belonging to Pd–X (X = N or C) and Pd–Pd, respectively. These results suggested the coexistence of single-atom Pd and Pd clusters, which were partly coordinated with N and/or C atoms of the residual C–N fragment derived from the organic components.^18^ This interaction was suggested to stabilize the Pd MSNCs/SAs, preventing them from aggregating.

NEXAFS spectroscopy was also employed to examine the N environment in the PCN⊂M samples using the N K-edge by taking TPT and MOP as references. As shown in Fig. 2c, the resonances of π* at 398.4 and 399.4 eV in pure TPT were assigned to nitrogen species in the form of pyridine (C–N (p)) and aromatic C–N–C coordination of tri-triazine, respectively. The π* resonance at 402.8 eV was attributed to charging effects or π excitations. The resonance of σ* at 405.2 eV was assigned to σ* transitions.19a,b Compared with TPT, the shift of the N 1s of PCN⊂M toward a higher binding energy indicated a decrease in the electron density of N.^20^ As compared to MOP, N 1s of PCN⊂M shifted to a lower binding energy. These two comparisons implied that the N electron density in PCN⊂M was between that in TPT and MOP. This indicated that there existed a Pd–N (pyridine type) in the PCN⊂M, but the bond strength was lower than that in MOP. This result was also in agreement with the Pd K-edge study, demonstrating the existence of positive coordination interaction between the Pd and N in the residual C–N fragments.

**Fig. 3:**
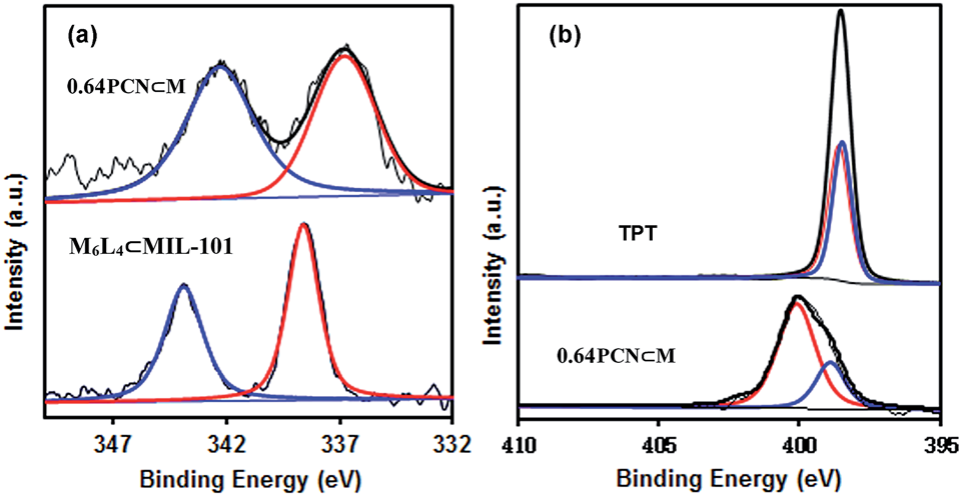
(a) Pd 3d and (b) N 1s XPS spectra of 0.64PCN⊂M, M_6_L_4_⊂MIL-101 and 2,4,6-tri(pyridin-4-yl)-1,3,5-triazine (TPT).

XPS measurements were also carried out to determine the palladium environment in 0.64PCN⊂M. As shown in Fig. 3a, 0.64PCN⊂M exhibited a Pd 3d_5/2_ band at around 336.9 eV and a Pd 3d_3/2_ band at around 342.4 eV, both of which were nearly 2 eV lower than those of the M_6_L_4_⊂MIL-101 (the M_6_L_4_⊂MIL-101 exhibited a Pd 3d_5/2_ band at 338.6 eV and a Pd 3d_3/2_ band at 343.9 eV). This result implied again that the Pd^II^ in M_6_L_4_ was reduced under the investigated conditions. The N 1s spectra of pure TPT showed two binding energies at around 398.3 eV and 398.6 eV, which were related to pyridine-type and triazine-type nitrogens, respectively.19*c* After assembly and reduction, the N environment of PCN⊂M underwent some changes. The N 1s bands of 0.64PCN⊂M were located at 398.9 and 400.2 eV, matching well with the character of pyridine-type (C–N) and triazine-type (C–N–C) nitrogens,19*c* and were 0.6 and 1.6 eV higher than those of pure **L**, respectively (Fig. 3b). The changes in binding energies suggested an electron interaction between N and Pd, which played an important role in preventing Pd from aggregating, as also demonstrated by the TEM, EXAFS and NEXAFS results.^21^ Furthermore, based on XPS data, the Pd content of 0.64PCN⊂M was calculated to be *ca.* 0.01%, which was much lower than the AAS result (0.64%). This result further confirmed that most of the Pd NPs were indeed encapsulated in the pores of MIL-101 since XPS is only able to detect the surface properties of few-atom layers.

The surface area and porosity of the hybrids were measured by N_2_ adsorption and desorption at 77 K. All PCN⊂M samples (Fig. S12†) showed similar isotherms to that of the parent MIL-101, indicating that the PCN⊂M materials still retained the porous structure of MIL-101. However, obvious decreases in the BET surface area and pore volume (Table S3†) were observed as compared with MIL-101, which were caused by the pore occupation by encapsulated Pd MSNCs/SAs. Reasonably, the BET surface area and pore volume of the PCN⊂M hybrids decreased gradually with an increase in Pd content.

Combining all these results, it can be confirmed that we have successfully fabricated and functionalized C–N decorated Pd MSNCs/SAs in the pores of MIL-101. The catalytic performance was then examined by taking hydrogenation as a model reaction due to its importance in the industrial production of aniline.^22^ Reactions were carried out under atmospheric pressure of H_2_ at room temperature. The parent MIL-101 showed almost no conversion (Table 1, entry 1), confirming the necessity of Pd to perform the hydrogenation. The influence of solvents on the catalytic performance was firstly examined using 0.64PCN⊂M as the catalyst. The results showed that CH_3_OH was the best solvent for this transformation under the investigated conditions (Table S4,† entries 2–6). Then the Pd loading effect was investigated. 0.64PCN⊂M showed the best catalytic performance with a TOF of 800 (Table 1, entry 4), which provided an almost complete conversion to aniline within only 5 min. 0.82PCN⊂M also showed excellent performance, giving a TOF of 776 (Table 1, entry 5). Obviously, this catalytic system represents an exceptional example for the quantitative hydrogenation of nitrobenzene under atmospheric pressure of H_2_ and room temperature, and it outperformed the most active Pd-based catalysts reported in the literature (Table S5†). More importantly, the catalyst could be recycled without any efficiency loss and Pd leaching for at least five runs (Fig. S13a†). To our delight, there was also no obvious Pd aggregation of the reused catalyst observed even after five runs (Fig. S13b†). These investigations showed that the PCN⊂M hybrids are stable catalysts for hydrogenation transformations, offering great potential in industrial applications under mild conditions.

**Table 1:** Hydrogenation of nitrobenzene to aniline over various catalysts^*a*^

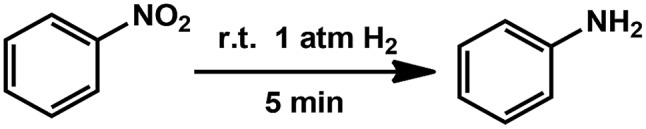				
Entry	Catalyst	Solvent	Yield (%)	TOF (h^–1^)
1	MIL-101	CH_3_OH	<1	—
2	0.33PCN⊂M	CH_3_OH	56	448
3	0.51PCN⊂M	CH_3_OH	76	655
4	0.64PCN⊂M	CH_3_OH	>99	800
5	0.82PCN⊂M	CH_3_OH	97	776
6	Pd/MIL-101	CH_3_OH	16	128
7	Pd/C	CH_3_OH	6	51

^*a*^Reaction conditions: nitrobenzene (0.1 mmol), 1 atm H_2_, room temperature, 5 min, 2 mL solvent, Pd/substrate = 1.5 mol%.

For comparison, a traditional impregnation method was also employed for the implantation of Pd into MIL-101, *i.e.*, Pd/MIL-101. Pd/MIL-101 was synthesized by soaking MIL-101 in an M_6_L_4_ aqueous solution, followed by H_2_ treatment. The loading efficiency was as low as 26% (Table S2†). The M_6_L_4_ was believed to be adsorbed on the MIL-101 surface as the M_6_L_4_ size was larger than the MIL-101 window, which was also verified by the TEM images, demonstrating a wide range of particle sizes (4–20 nm) and severe aggregation (Fig. S14†). As a result, Pd/MIL-101 showed much lower catalytic activity than PCN⊂M under the same conditions, affording only 16% yield (TOF: 128) of aniline (Table 1, entry 6). The TOF of PCN⊂M was also much higher than that of commercial 5% Pd/C (TOF: 51, Table 1, entry 7). Considering the similar active sites but different structures of PCN⊂M, Pd/MIL-101 and Pd/C, it is reasonable to suggest that the outstanding catalytic activity over PCN⊂M can be directly ascribed to its highly exposed Pd atoms and the positive synergistic effect between Pd MSNCs/SAs and C–N fragments, which was also verified by the EXAFS and XPS results.

The PCN⊂M also showed excellent catalytic performance towards the selective hydrogenation of biomass-derived furfural (FFA) to cyclopentanone (CPO). Due to the abundance of FFA sources and high added-value of CPO, the selective transformation of FFA to CPO has received increasing interest in recent years.^23^ Even though various catalyst systems for FFA hydrogenation have been developed, the harsh reaction conditions (such as ultrahigh H_2_ pressure) and low selectivity are still a significant bottleneck which hinders their practical application (Table S6†). Consequently, the need to design a highly selective but mild catalytic system towards sustainable development is urgent. As expected, our PCN⊂M system also showed unprecedented catalytic performance in aqueous hydrogenation of FFA to CPO under a low H_2_ pressure. The parent MIL-101 gave essentially no reactivity (Table 2, entry 1). In sharp contrast, the 0.64PCN⊂M catalyst showed amazing performance with both ultra-high conversion (>99%) and selectivity (>99%) (Table 2, entry 4). Other PCN⊂M catalysts with different loadings also showed good performance with high selectivity (Table 2, entries 2, 3 and 5). Different reaction temperatures were subsequently screened (Fig. S15†). As the temperature increased, the CPO yield increased gradually. It is worth mentioning that even when the reaction temperature was 200 °C, the CPO selectivity was still >99% with few over-hydrogenated products (*i.e.*, CPL).

**Table 2:** Hydrogenation of FFA to CPO over various catalysts^*a*^

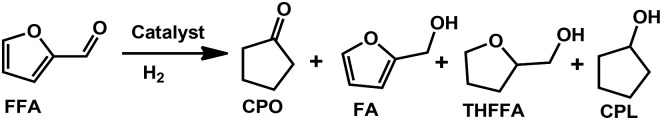				
Entry	Catalyst	Conv. (%)	CPO sel. (%)	CPL sel. (%)
1	MIL-101	—	—	—
2	0.33PCN⊂M	85	>99	<1
3	0.51PCN⊂M	91	>99	<1
4	0.64PCN⊂M	>99	>99	<1
5	0.82PCN⊂M	94	>99	<1
6	Pd/MIL-101	46	83	17
7	Pd/C	28	71	29

^*a*^Reaction conditions: FFA (0.52 mmol), Pd/FFA = 1.15 × 10^–3^, water (4 mL), 180 °C, 0.8 MPa H_2_, 24 h.

We also investigated the effect of H_2_ pressure on CPO yield (Fig. S16†). Under the investigated conditions, the reaction could also proceed even when the pressure was as low as 1 atm (Fig. S17†), which was much lower as compared to other reported systems (Table S6†). Generally speaking, selectivity would decrease with increasing H_2_ pressure. To our delight, the CPO yield over 0.64PCN⊂M was still excellent (>99%) even when the pressure reached 1.2 MPa.

In general, the stability and reusability of MOF catalysts can be particularly challenging under aqueous reaction conditions at moderate to high temperatures. However, the PCN⊂M could be used up to 5 times without any significant decrease in catalytic performance, which was checked by terminating the reaction at 12 h (Fig. S18†). Furthermore, no metal leaching was observed by AAS analysis of the liquid phase after the reaction. All these investigations indicated the robustness and effectiveness of this newly developed catalytic system.

## Conclusions

In summary, we have developed a facile strategy to fabricate highly dispersed C–N-decorated MSNCs/SAs that were encapsulated in the pores of a MOF. The obtained PCN⊂M hybrids contained both Pd sub-nanoclusters and Pd single atoms, which were stabilized by the *in situ* formed C–N fragments and the confinement effect offered by the MOF pores. The confined C–N-decorated MSNCs/SAs exhibited superior catalytic activity and stability in important catalytic reactions. This approach offers a versatile approach for the encapsulation of a broad range of similar MSNCs/SAs into MOF pores and broadens the library of decorated MSNCs/SAs. Studies aimed at extending this strategy for the encapsulation of other types of MSNCs/SAs into MOFs for advanced catalysis applications are currently underway in our laboratory.

## Conflicts of interest

There are no conflicts to declare.

## Supplementary Material

Supplementary information
