# Supplementary material for: Encapsulation of C–N-decorated metal sub-nanoclusters/single atoms into a metal–organic framework for highly efficient catalysis
Source: Chem Sci. 2018 Sep 26;9(48):8962–8. doi: 10.1039/c8sc03549k (PMC6296299; doi:10.1039/c8sc03549k)
Supplement: Supplementary file 1 [file SC-009-C8SC03549K-s001.pdf]

Electronic Supplementary Material (ESI) for Chemical Science.
This journal is © The Royal Society of Chemistry 2018

Supporting Information

Encapsulation of C-N-decorated metal sub-nanoclusters/single atoms into a Metal-organic framework for highly-efficient catalysis

Xuan Qiu,[#] Jianmin Chen,[#] Xinwei Zou, Ruiqi Fang, Liyu Chen, Zhijie Chen, Kui Shen* and Yingwei Li*

State Key Laboratory of Pulp and Paper Engineering, School of Chemistry and Chemical Engineering, South China University of Technology, Guangzhou 510640, China.

#These authors contributed equally to this work

E-mail: *cekshen@scut.edu.cn (K.S.), liyw@scut.edu.cn (Y.L.)*

This PDF file includes:

Experimental Section

Figures S1 to S18

Tables S1 to S6

Experimental Section

Chemicals

All chemicals were purchased from commercial sources and used without further purification. All solvents were analytical grade and distilled prior to use.

Synthesis of M_6L_4 (M: (en)Pd(NO_3)$_2$; L: 2,4,6-tri(pyridin-4-yl)-1,3,5-triazine) [S1]

M_6L_4 was prepared according to the literature report. [S1] **M** (60 mg, 0.206 mmol), **L** (43 mg, 0.137 mmol), and 10 mL water were stirred at room temperature for 24 h. The mixture was filtered and the filtrate was concentrated with evaporator to obtain the light-yellow solid.

Synthesis of MIL-101[S2]

$Cr(NO_3)_3 \cdot 9H_2O$ (2.00 g, 5 mmol), HF (48 wt%, 5 mmol), and terephthalic acid (0.823 g, 5 mmol) were added into 24 mL deionized water. After heating at 220 °C for 8 h, the mixture was cooled first to 150 °C in 1 h, and then slowly to room temperature in 12 h. The green MIL-101 powder was isolated from the solution and washed with deionized water and ethanol, and then soaked in ethanol (95% EtOH with 5% water) at 80 °C for 24 h. The obtained solid was finally dried overnight at 150 °C under vacuum.

Synthesis of $M_6L_4 \subset$MIL-101

6.3 mg **L** was added to 8 mL n-hexane. After sonicating for 2 h, 310 mg activated MIL-101 was added. The suspension was stirred at room temperature for 20 h, to which a small amount of aqueous **M** (7 mg) solution (0.40 mL) was added dropwise under vigorous stirring. Afterwards, the suspension was stirred for another 72 h. The

obtained solid was washed with water, and activated by heating at 150 °C for 12 h under dynamic vacuum.

Synthesis of PCN⊂M

The M_6L_4⊂MIL-101 hybrids were treated at 250°C under H_2 flow for 2 h. The heating rate and cooling rate were 0.5 °C/min. The obtained hybrids were washed with H_2O and ethanol, and then dried at room temperature. PCN⊂M hybrids with different Pd contents were synthesized according to the above protocol by varying the amount of precursors. The obtained hybrids were named as XPCN⊂M (X = 0.33, 0.51, 0.64, 0.82), where X represents the actual Pd weight percentage, as measured by atomic absorption spectrometry (AAS). For 0.33PCN⊂M, 0.51PCN⊂M, 0.64PCN⊂M and 0.82PCN⊂M, the added **L** precursor was 3.0 mg, 4.7 mg, 6.3 mg and 7.4 mg, and the **M** mass was 3.5 mg, 5.2 mg, 7 mg and 8.6 mg, respectively. The theoretical Pd loading, the actual Pd loading and the doping efficiecy were shown in Table S2.

Synthesis of Pd/MIL-101

310 mg MIL-101 was soaked in M_6L_4 aqueous solution (33 mg M_6L_4 in 8 mL H_2O). After stirring for 24 h, the suspension was filtered and washed with H_2O for several times. The obtained solid was washed with water, activated by heating at 150 °C for 12 h under dynamic vacuum, and then heated at 250 °C in H_2 for 2 h. The heating rate and cooling rate were both 0.5 °C/min. The theoretical Pd loading, the actual Pd loading and the doping efficiecy were shown in Table S2.

Nitrobenzene hydrogenation reaction

Typically, nitrobenzene (0.1 mmol) and catalyst (0.0015 mmol Pd) were added to a Schlenk tube. Ethanol (2 mL) was then added under hydrogen at room temperature. Then an hydrogen balloon was connected to the Schlenk tube. The reaction mixture was stirred at room temperature for the desired time. Afterwards, the reaction liquid was collected by centrifugation. the solid catalyst was washed with ethyl acetate (3 × 5 mL). The liquid was all collected and concentrated in vacuo. The crude product was quantified by GC-MS analysis.

Heterogeneity of the catalyst

To verify whether the catalysis of 0.64PCN⊂M was truly heterogeneous, the solid catalyst was filtered from the reaction solution after 2 min (Con. 52%) in nitrobenzene hydrogenation reaction. The reaction was continued with the filtrate in the absence of solid catalyst for an additional 5 min. The solution in the absence of solid did not exhibit any further reactivity. The results demonstrated that the reaction proceeded mostly on the heterogeneous surface.

Catalytic hydrogenation of furfural

Typically, FFA (0.52 mmol), catalyst (Pd/FFA = 1.15 * 10^{-3}) and water (4 mL) were added to a 10 mL stainless steel autoclave equipped with a magnetic stirrer, a pressure gauge, and an automatic temperature controller. Then the autoclave was sealed and purged with hydrogen at low pressure for several times to remove air. Then the autoclave was purged with H_2 for the desired pressure and then set to the

desired temperature. After reaction, the autoclave was cooled to room temperature and the reaction liquid was collected by centrifugation. The solid catalyst was washed with ethyl acetate (3 × 5 mL). The liquid was all collected and concentrated in vacuo. The crude product was quantified by GC-MS analysis.

The conversions and selectivities were calculated based on the moles of furfural. The conversion of furfural (mol%) and cyclopentanone selectivity (mol%) were calculated using the following equations:

$$\text{Furfural conversion} = \left(1 - \frac{\text{Moles of furfural}}{\text{Moles of furfural loaded}}\right) \times 100\%$$

$$\text{Cyclopentanone yield} = \frac{\text{Moles of cyclopentanone}}{\text{Moles of furfural loaded}} \times 100\%$$

Recycling of the catalysts

The recyclability of the catalysts was tested for the nitrobenzene hydrogenation reaction and the catalytic hydrogenation of furfural under the investigated reaction conditions as described above except using the recovered catalysts. Each time, the catalyst was separated from the reaction mixture by centrifugation at the end of catalytic reaction, thereafter, washed with ethyl acetate and ethanol, dried at room temperature. The catalyst powder was reused as catalyst for a new run.

Materials characterization

BET surface area measurements were performed with N_2 adsorption/desorption isotherms at 77 K on a Micromeritics ASAP 2020M instrument. Prior to analysis, the samples were degassed at 150 °C for 12 h. Powder X-ray diffraction patterns of the

samples were recorded on a Bruker D8 ADVANCR using Cu Kα radiation (40 kV, 40 mA).

The size and morphology of the materials were determined by a scanning electronic microscope (SEM, **Merlin** from **Zeiss**) equipped with an energy dispersive X-raydetector (EDS, X-MaxN20 from Oxford), Transmission Electron Microscopy (TEM) (JEM-2100F, JEOL, Tokyo, Japan) as well as cold field-emission spherical-aberration corrected TEM (Cold FEG Cs corrected TEM) (JEM-ARM200F, Japan) were operated at an accelerating voltage of 200 kV. Energy dispersive X-ray spectroscopy (EDX) was performed to locate elemental distribution of Pd, Cr and N with an SDD-type EDX detector. The attainable energy-resolution of the EDX detector is 130 eV. The identification and quantitation of products were performed on a GC-MS spectrometer (Agilent Technologies 7890B-5977A equipped with a 0.25 mm × 30 m HP-5MS capillary column).

The Pd contents in the samples were measured quantitatively by atomic absorption spectroscopy (AAS) on a HITACHI Z-2300 instrument. XPS spectra were recorded on ESCALAB 250Xi from Thermo Fisher-VG Scientific using Mono Al Kα as the X-ray source. Elemental analysis was performed on an Elementar Vario EL III equipment by weighing samples of 0.2-0.3 mg and packing with aluminium foil for the measurement. All materials were degassed at 150 °C for 12 h before measurement. TGA-DSC was performed on a Q600 SDT from TAINC under N_2 atmosphere. H_2-TPR measurements were performed on DAS-7200. The samples were pre-treated under an Ar atmosphere at 150 °C for 1 h to clean the surface, followed by cooling

down to room temperature. The flow of 10% H_2/Ar (50 mL/min) was then switched into the system, and the samples were heated up to 800 °C from room temperature at a rate of 10 °C/min.

The X-ray absorption data at the Pd *K* - edge of the samples were recorded at room temperature in transmission mode and in the fluorescent mode with silicon drift fluorescence detector at beam line BL14W1 of the Shanghai Synchrotron Radiation Facility (SSRF), China. The station was operated with a Si(311) double crystal monochromator. During the measurement, the synchrotron was operated at energy of 3.5 GeV and a current between 150-210 mA. The photon energy was calibrated with the first inflection point of Pd K-edge in Pd metal foil.

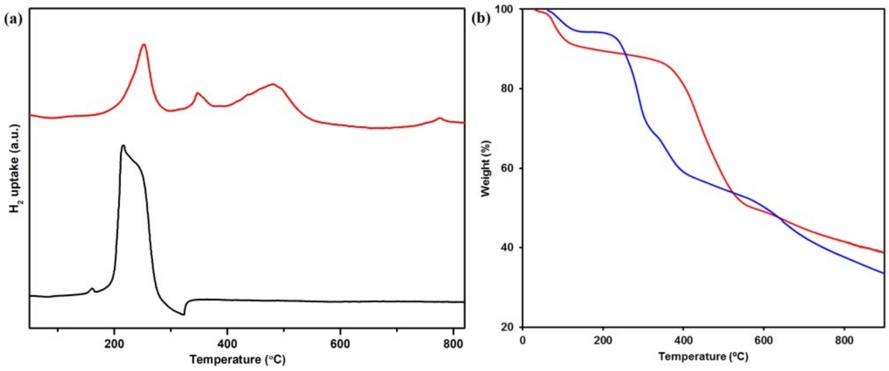

Fig. S1 (a) TPR patterns of M_6L_4 (black curve) and M_6L_4⊂MIL-101 (red curve); (b) TGA results of MIL-101 (red curve) and M_6L_4 (blue curve).

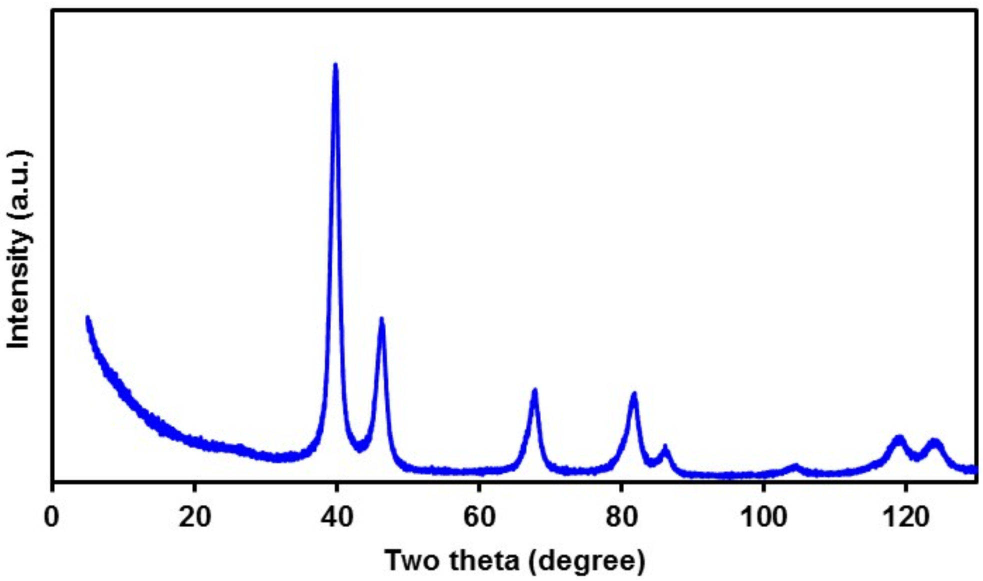

Fig. S2 PXRD patterns of Pd/C-N.

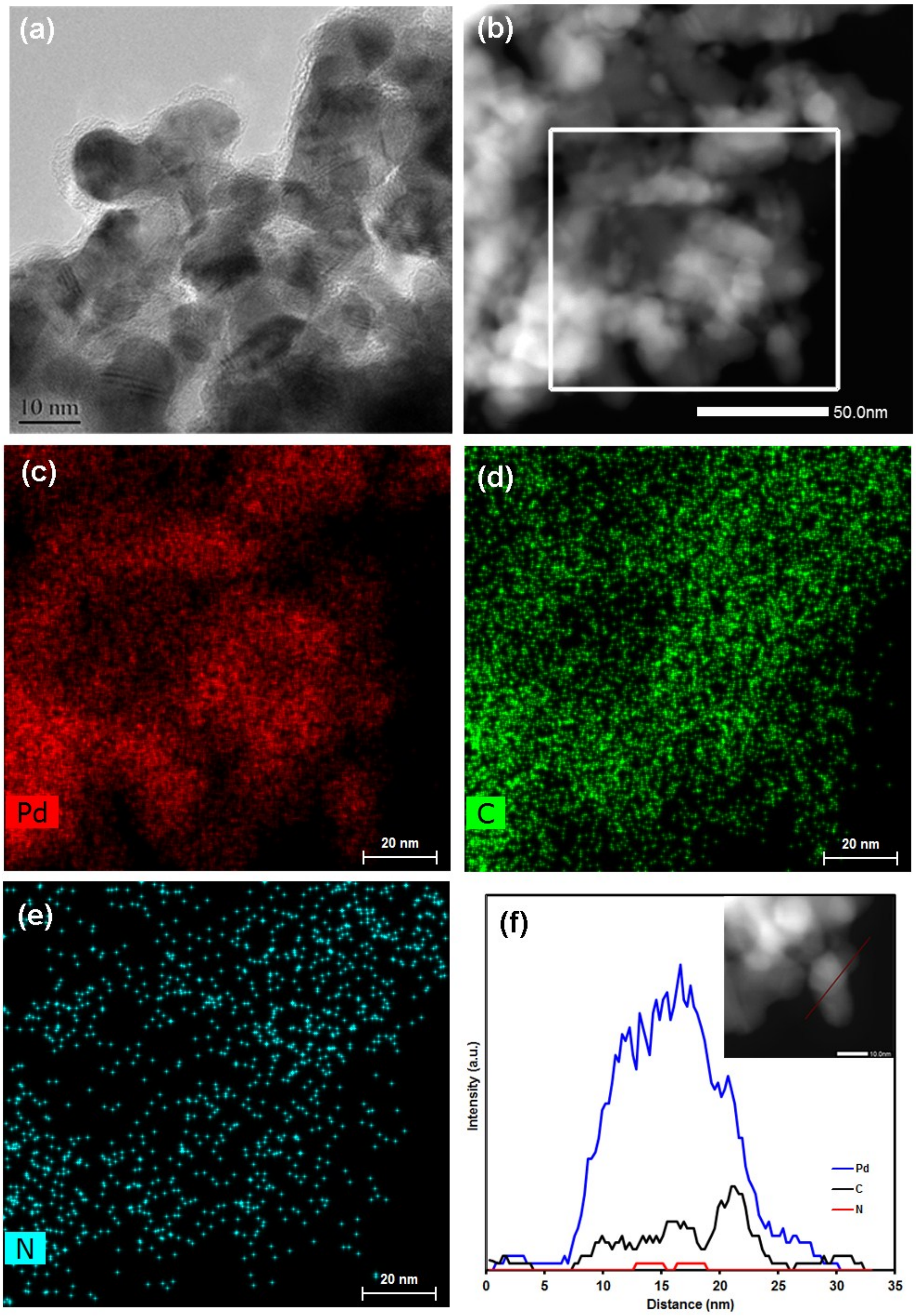

Fig. S3 (a) TEM image of Pd/C-N. (b) HAADF-STEM image of Pd/C-N and (c-e) corresponding EDX elemental mapping images of Pd, C, and N. (f) Elemental line-scanning spectra of Pd/C-N along the direction marked by a red line (inset).

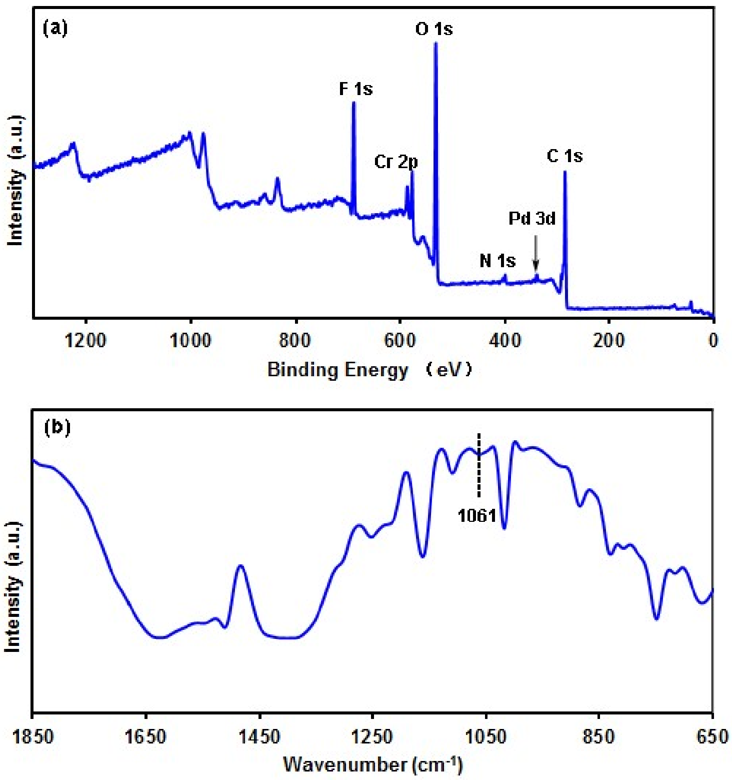

Fig. S4 (a) XPS and (b) FT-IR spectrum of the $M_6L_4 \subset$MIL-101.

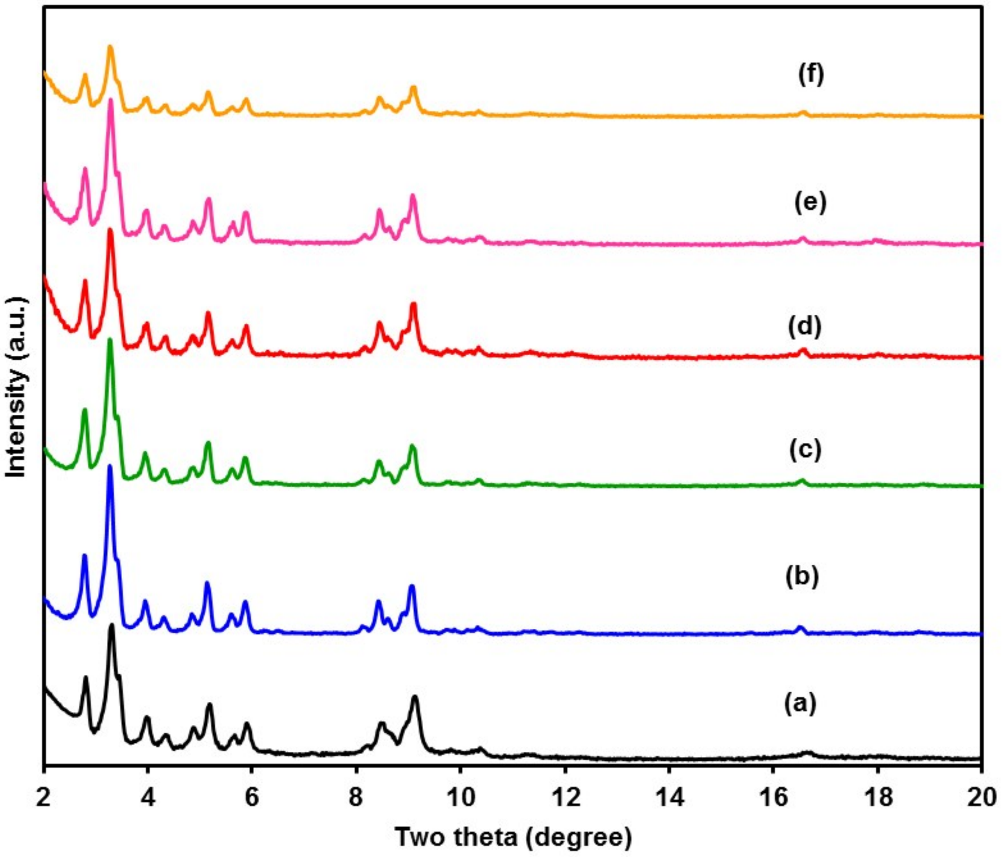

Fig. S5 PXRD patterns of (a) MIL-101, (b) 0.82PCN⊂M, (c) 0.64PCN⊂M, (d) 0.51PCN⊂M, (e) 0.33PCN⊂M and (f) the reused 0.64PCN⊂M.

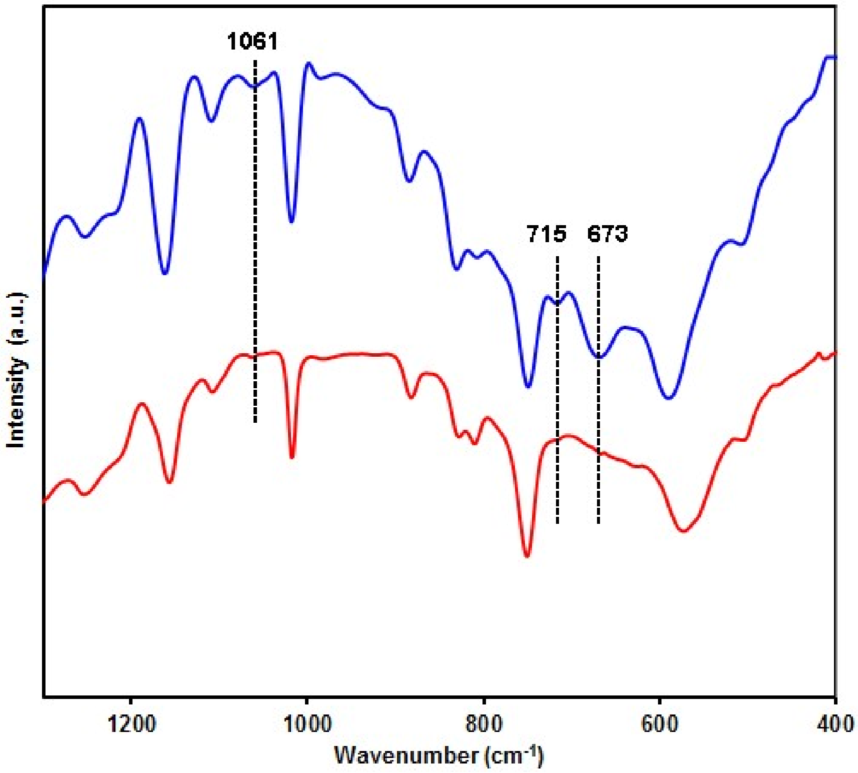

Fig. S6 FT-IR spectra of the M_6L_4⊂MIL-101 (blue line) and 0.64PCN⊂M (red line).

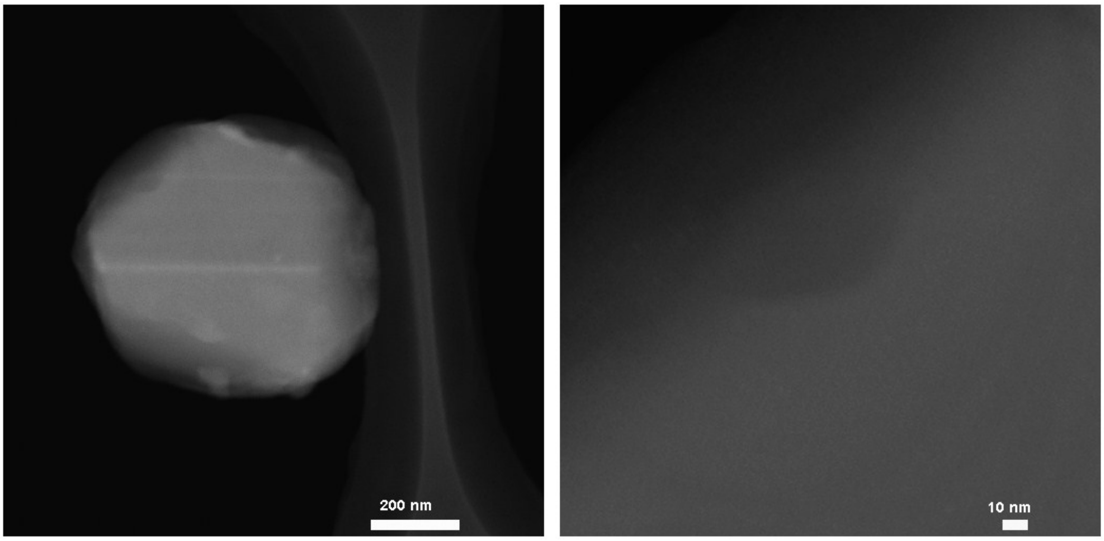

Fig. S7 HAADF-STEM images of $M_6L_4 \subset$MIL-101.

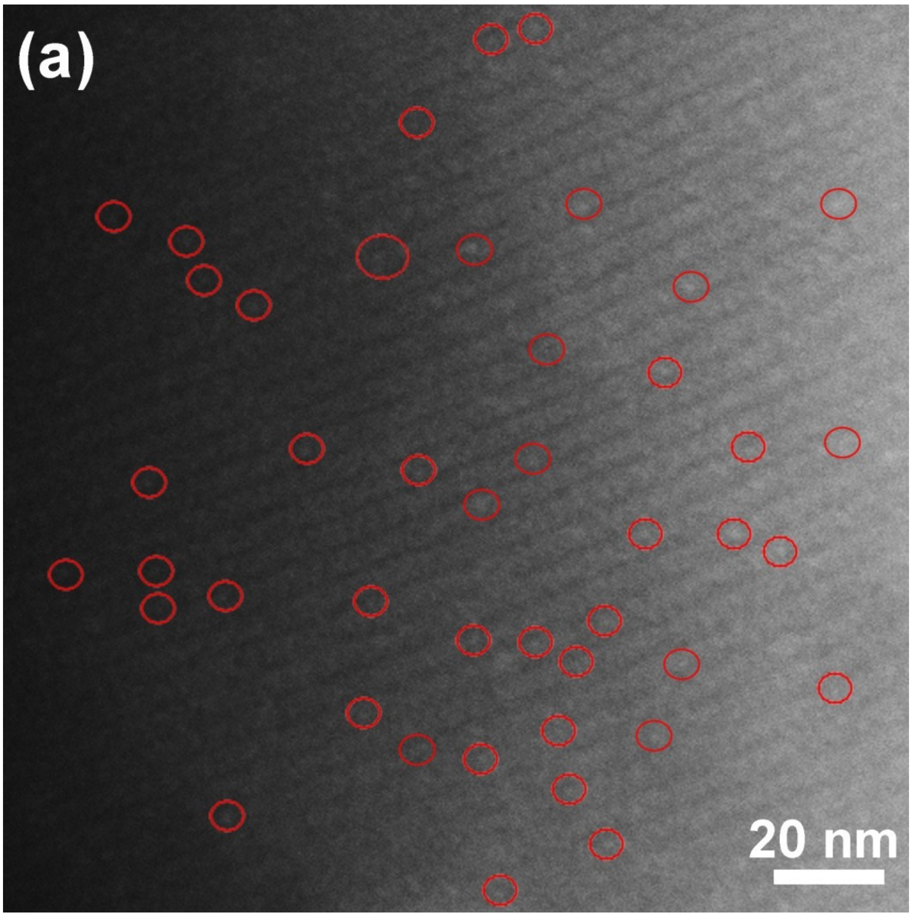

Fig. S8 (Enlarged Fig. 1a) HAADF-STEM images of ultrathin cuts from 0.33PCN⊂M.

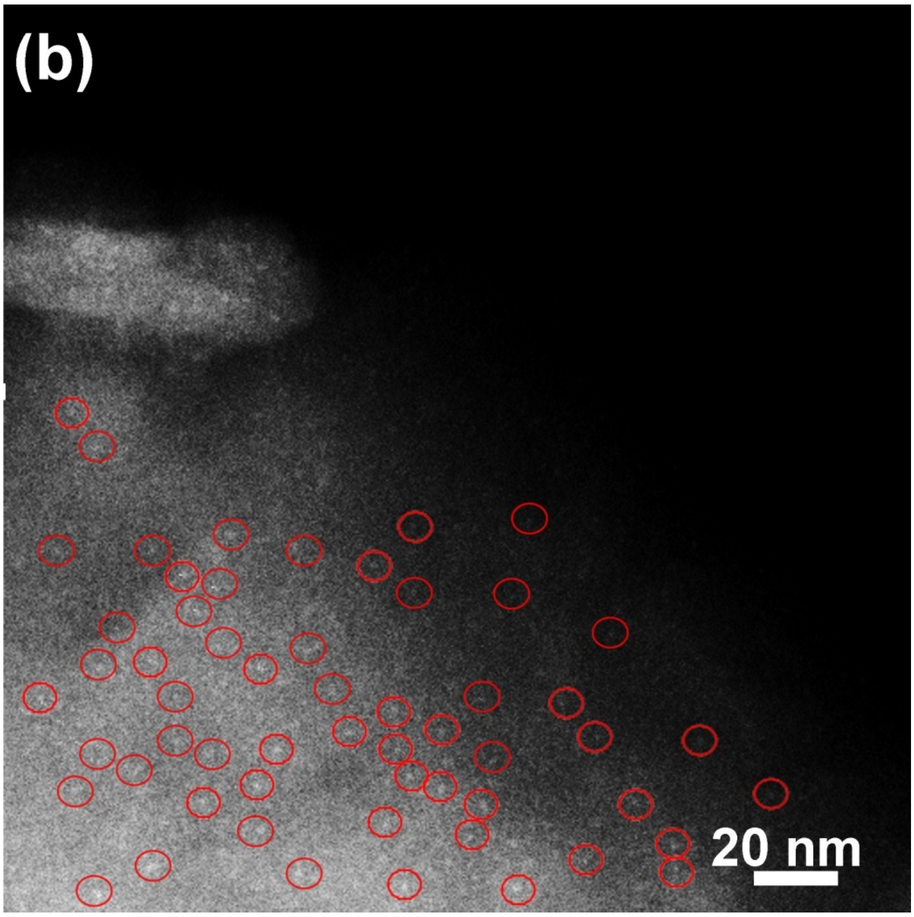

Fig. S9 (Enlarged Fig. 1b) HAADF-STEM images of ultrathin cuts from 0.51PCN⊂M.

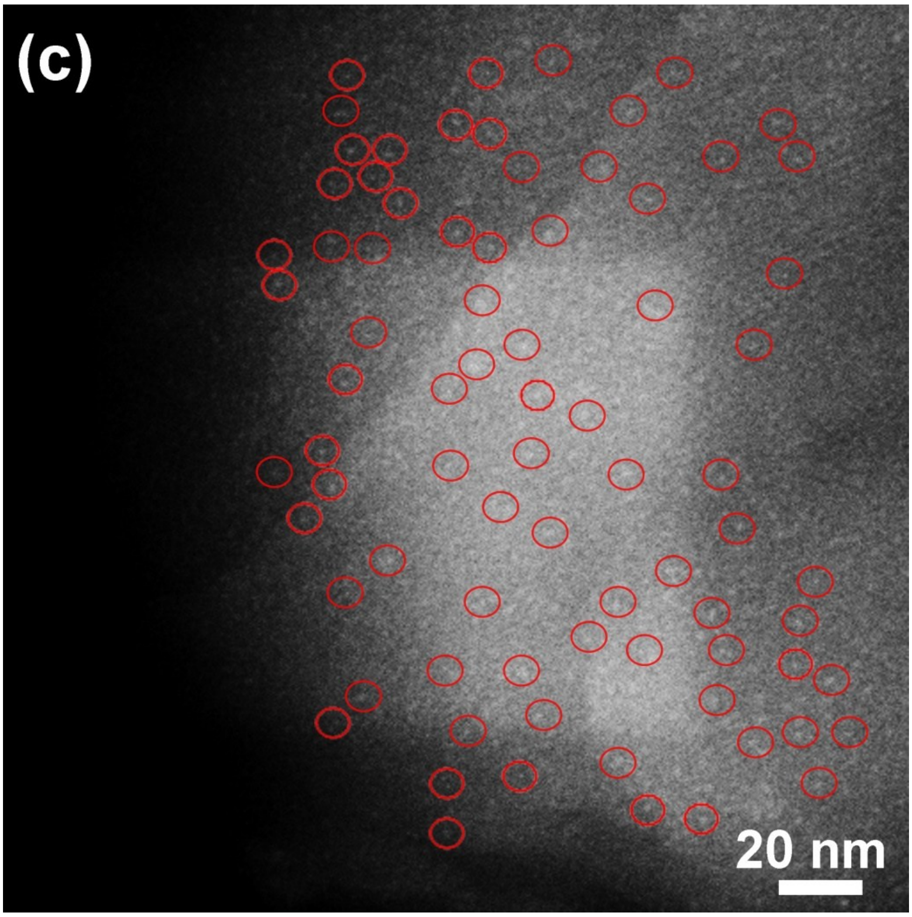

Fig. S10 (Enlarged Fig. 1c) HAADF-STEM images of ultrathin cuts from 0.64PCN⊂M.

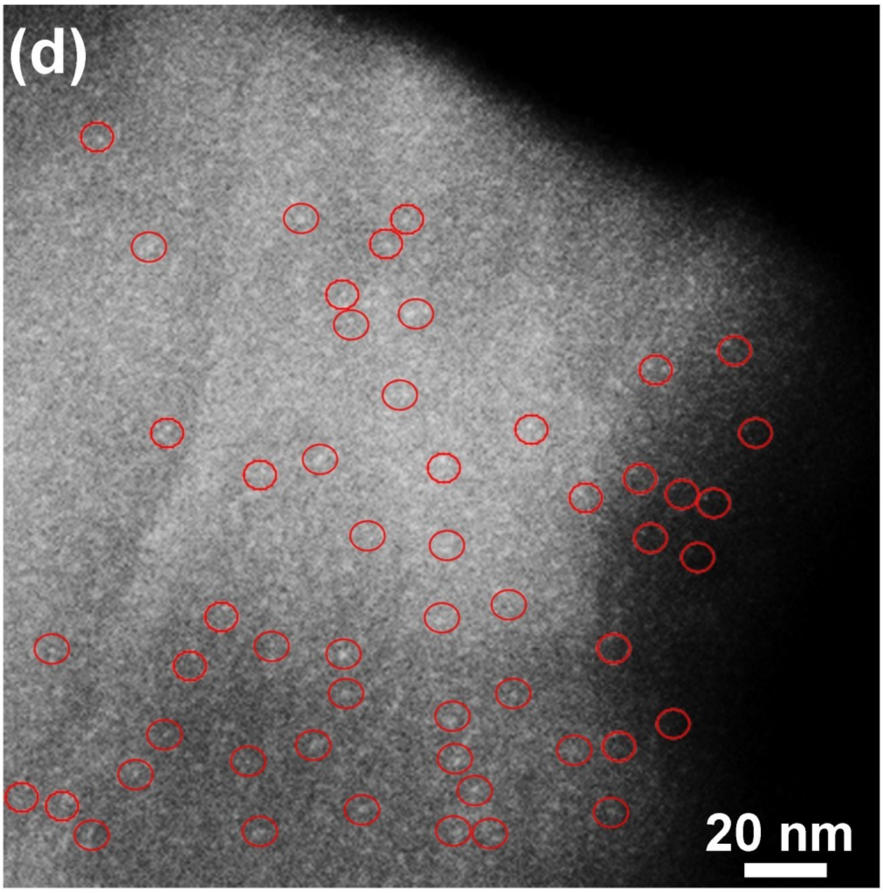

Fig. S11 (Enlarged Fig. 1d) HAADF-STEM images of ultrathin cuts from 0.82PCN⊂M.

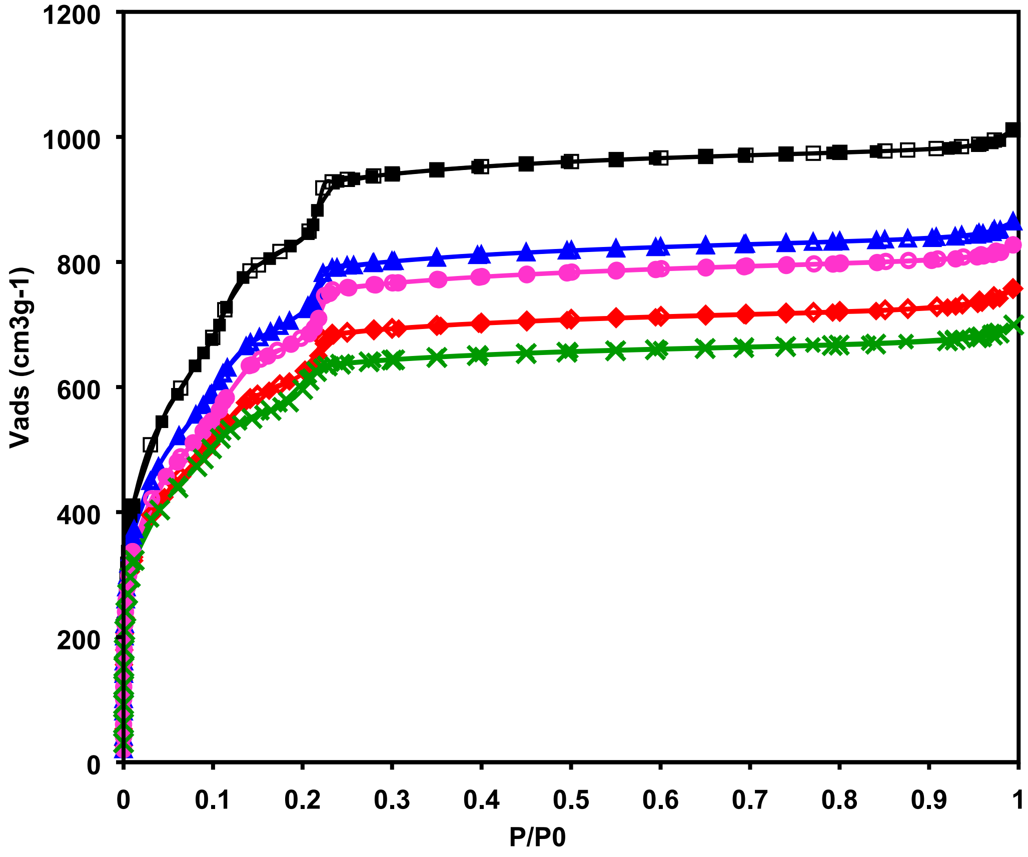

Fig. S12 Nitrogen adsorption/desorption isotherms at 77 K for the MIL-101 and PCN⊂M hybrids: □ for parent MIL-101, △ for 0.33PCN⊂M, ○ for 0.51PCN⊂M , ◇for 0.64PCN⊂M, ×for 0.82PCN⊂M.

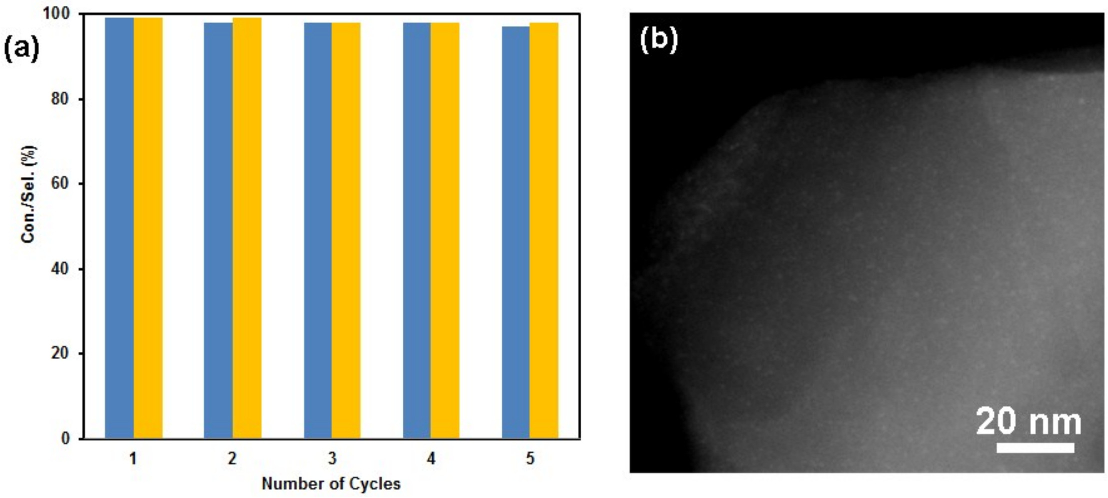

Fig. S13 (a) The conversion and selectivity of nitrobenzene hydrogenation over the 0.64PCN⊂M, blue for conversion, and yellow for selectivity. (b)The STEM image of the reused 0.64PCN⊂M catalyst after the 5th run.

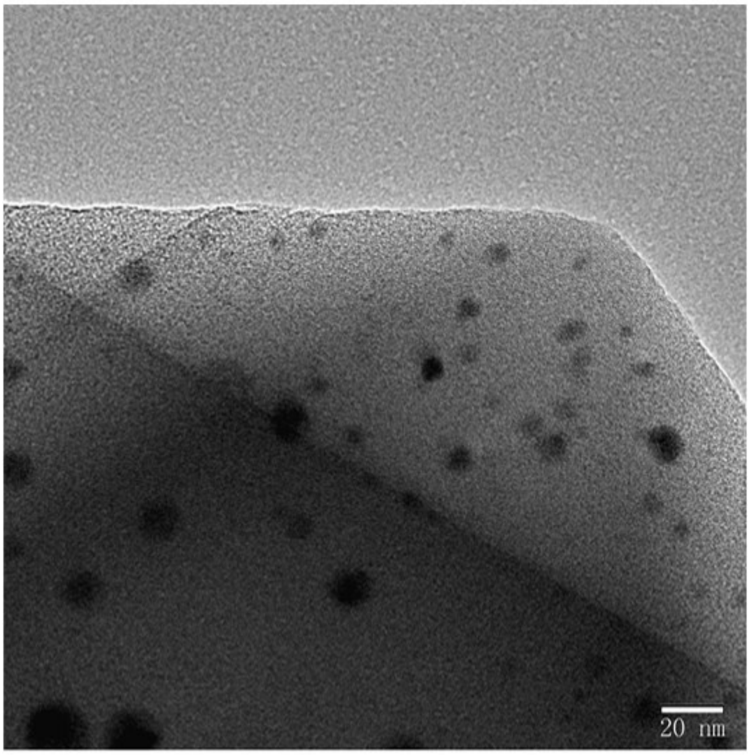

Fig. S14 TEM image of Pd/MIL-101.

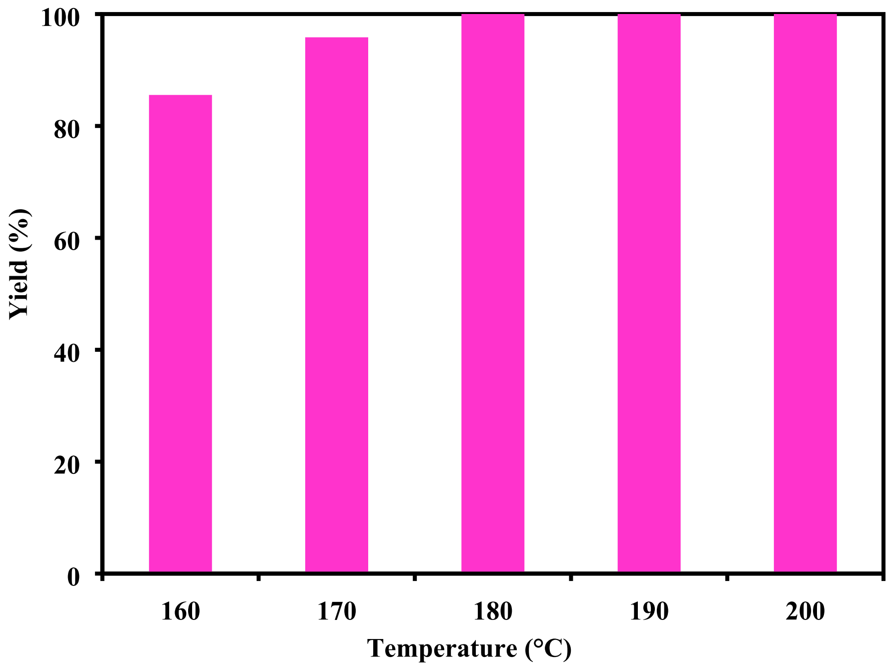

Fig. S15 Effect of temperature on CPO yield over 0.64PCN⊂M. Reaction conditions: FFA (0.52 mmol) , Pd/FFA = 1.15*10^{-3}, water (4 mL), 0.8 MPa H_2, 24 h.

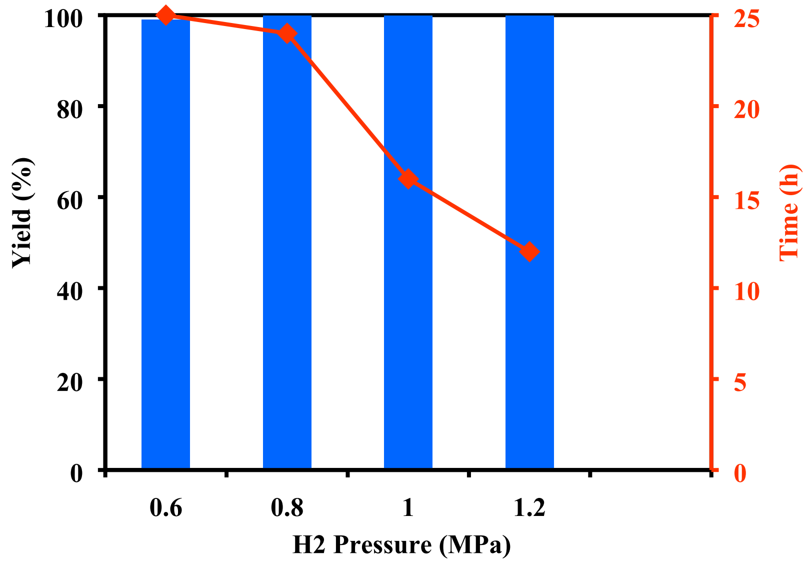

Fig. S16 Effect of hydrogen pressure on CPO yield over 0.64PCN⊂M. Reaction conditions: FFA (0.52 mmol), Pd/FFA = 1.15*10^{-3}, water (4 mL), 180 °C.

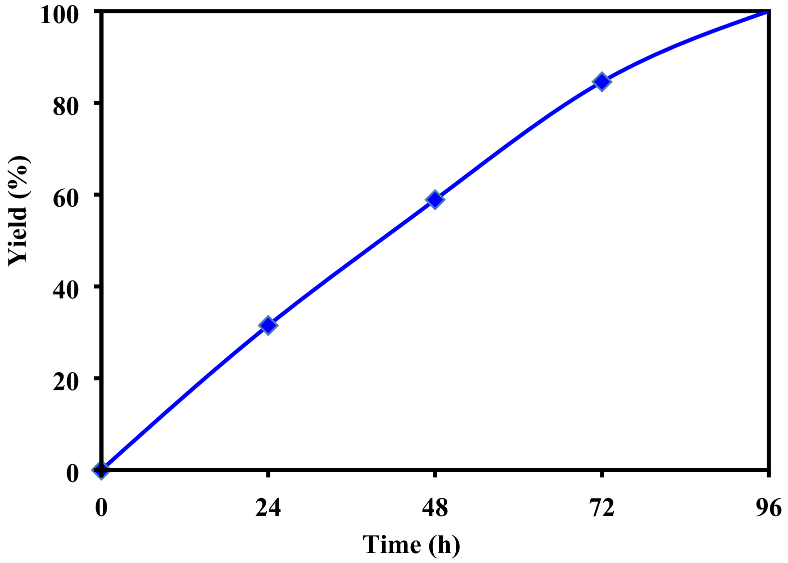

Fig. S17 CPO yield as a function of reaction time. Reaction conditions: FFA (0.52 mmol), Pd/FFA = 1.15*10^{-3}, water (4 mL), 180 ^{o}C, 1 atm H_2.

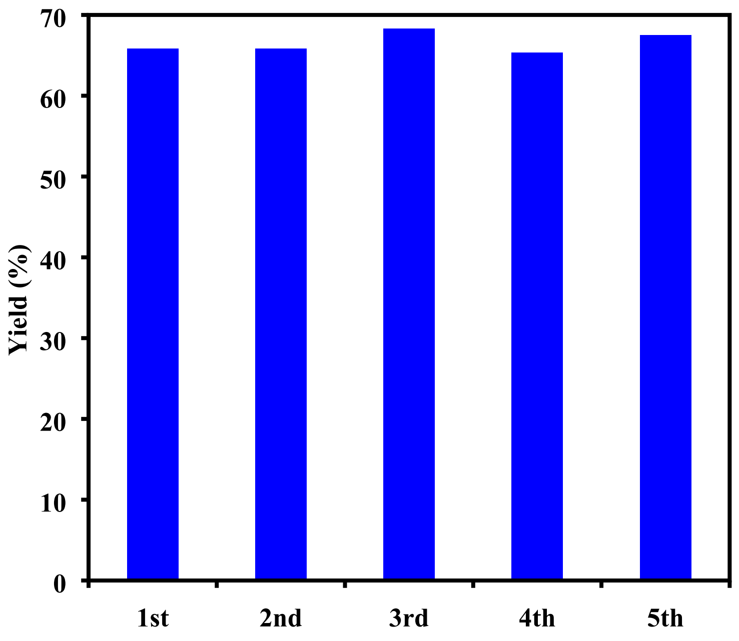

Fig. S18 Reuse of the 0.64PCN⊂M catalyst in FFA hydrogenation. Reaction conditions: FFA (0.52 mmol), Pd/FFA = 1.15*10^{-3}, water (4 mL), 180 ^{o}C, 0.8 MPa H_2. Each reaction was terminated at 12 h.

Table S1. The element contents of the Pd/C-N hybrids derived from M_6L_4.

Sample	C content (wt%)[a]	N content (wt%)[a]	H content (wt%)[a]	Pd content (wt%)[b]
Pd/C-N	26.40	8.14	3.20	56.90

[a] Measured by elemental analysis.

[b] Measured by AAS.

Table S2. The actual Pd contents in the PCN⊂M hybrids measured by AAS.

Sample	Theoretical Pd content (%)	Actual Pd content (%)	Doping percentage (%)
0.33PCN⊂M	0.40	0.33	83
0.51PCN⊂M	0.60	0.51	85
0.64PCN⊂M	0.80	0.64	80
0.82PCN⊂M	1.00	0.82	82
Pd/MIL-101	2.20	0.57	26

Table S3. BET surface areas and pore volumes of the materials.

Sample	S_{BET} ($m^2\ g^{-1}$)	Pore volume ($cm^3\ g^{-1}$)
MIL-101	2988	1.56
0.33PCN⊂M	2559	1.33
0.51PCN⊂M	2424	1.28
0.64PCN⊂M	2221	1.17
0.82PCN⊂M	2112	1.08

Table S4. Hydrogenation of nitrobenzene (**1a**) to aniline (**3a**)[a]

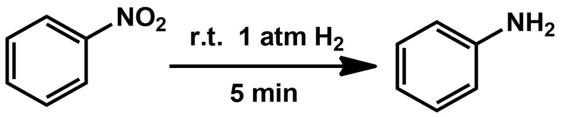

Entry	Catalyst	Solvent	Yield (%)	TOF (h^{-1})
1	MIL-101	CH_3OH	<1	-
2	0.64PCN⊂M	Toluene	76	614
3	0.64PCN⊂M	CH_3OH	>99	800
4	0.64PCN⊂M	CH_3CH_2OH	60	480
5	0.64PCN⊂M	H_2O	88	704
6	0.64PCN⊂M	Ethyl acetate	24	192
7	0.82PCN⊂M	CH_3OH	97	776
8	0.33PCN⊂M	CH_3OH	56	448
9	0.51PCN⊂M	CH_3OH	76	655
10	Pd/MIL-101	CH_3OH	16	128
11	Pd/C	CH_3OH	6	51

[a] Reaction conditions: nitrobenzene (0.1 mmol), 1 atm H_2, room temperature, 5 min, 2 ml solvent, Pd/substrate = 1.5 mol%.

Table S5. Summary of the catalytic performance of typical Pd-based catalysts for hydrogenation of nitrobenzene.

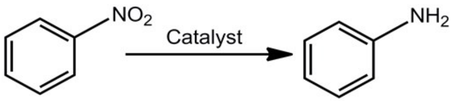

Catalyst	Pd loading (wt%)	Solvent, *T* (^{o}C)	H_2 pressure (kPa)	Time (h)	Conversion (%)	Selectivity (%)	TOF[b] (h^{-1})	Note
0.64PCN⊂M	0.64	methanol, r.t.	101.3	0.083	＞99	＞99	800	this work[a]
0.82PCN⊂M	0.82	methanol, r.t.	101.3	0.083	97	＞99	776	this work[a]
0.33PCN⊂M	0.33	methanol, r.t	101.3	0.083	56	＞99	448	this work[a]
0.51PCN⊂M	0.51	methanol, r.t	101.3	0.083	76	＞99	655	this work[a]
Pd@NKZPDB-1	0.03	methanol, r.t	101.3	3	89.2	100	129	Ref[3]
1 at% Pd-coated Ni	Unknown	methanol, r.t	101.3	6	100	Unknown	367	Ref[4]
Pd/PVDF-EC	0.9	water, r.t	Unknown	2.5	98	100	92	Ref[5]
Pd/PEG catalyst	3.75	EtOH, r.t	101.3	2(3)	93(100)	93(100)	71(83)	Ref[6]
Pd^{o}-in-UiO-66	1.0	THF, r.t.	101.3	3	100	99	165	Ref[7]
HMMS-NH_2-Pd	2.34	EtOH, r.t	101.3	1	100	＞99	100	Ref[8]
Pd/C	10	EtOH, r.t	101.3	2	100	＞99	50	Ref[8]
Fe_3O_4@SiO_2-SH-Pd	3.81	EtOH, r.t	101.3	0.83	100	＞99	168	Ref[9]
Pd(0)-CF	0.73	EtOH, 35±2°C	1000	2.67	52	＞99	162	Ref[10]
Pd(0)-$EGCG_{0.2}$-CF	1.1	EtOH, 35±2°C	1000	2.67	98	＞99	306	Ref[10]
Pd/γ-Al_2O_3	1.0	EtOH, 35±2°C	1000	2.67	100	＞95	312	Ref[10]
Pd/SiO_2	1.0	EtOH, 35±2°C	1000	2.67	88	＞95	275	Ref[10]
Pd/γ-Al_2O_3/Fe_3O_4/SiO_2	1.0	EtOH, 20°C	200	0.5	99	92	206	Ref[11]
Pd@Fe_3O_4	Unknown	EtOH, r.t	101.3	1	＞99	—	49	Ref[12]
(Pd/bpy)10	Unknown	EtOH, 24°C	122	5.3	＞99	—	225	Ref[13]
Fe_3O_4-NH_2–Pd	8.43	EtOH, r.t	101.3	0.75	＞99	＞99	83	Ref[14]
Pd/γ-Fe_2O_3/urea-MCF	5	MeOH, 25°C	689	16	＞98	＞98	6	Ref[15]

Pd NPs	Unknown	MeOH, 25°C	101.3	4	Yield: 98	252	Ref[16]
Pd/Fe_3O_4	2.3	EtOH, 25-40°C	101.3	2-3	Yield: 99	50	Ref[17]
Pd /SiO_2	Unknown	EtOH, r.t	101.3	1	Yield:27.2	24	Ref[18]
HMMS-salpr-Pd	5.12	EtOH, r.t	101.3	1	Yield:＞99	106	Ref[19]

[a] *Reaction conditions: nitrobenzene (0.1 mmol), 1 atm H_2, room temperature, 5 min, 2 ml solvent, Pd/substrate = 1.5 mol%.*

[b] *TOF of the catalyst was calculated as the number of moles of product per mol of Pd per hour.*

Table S6. Summary of the reaction results of FFA hydrogenation to CPO over different catalysts.

Catalyst	Metal loading (wt%)	H_2 Pressure (MPa)	T (°C)	Note
Co/ZrO_2-La_2O_3	8.8	2	160	Ref [S20]
$CuNi_{0.5}$@C	unkown	5	130	Ref [21]
Au/TiO_2-A	0.1	4	160	Ref [22]
5%Pd-10%Cu/C	1	3	160	Ref [23]
Ru/MIL-101	3	4	160	Ref [24]
Cu-Co-CP-500	5	2	170	Ref [25]
CuZnAl-500-0.5		4	150	Ref [26]
Cu-Ni-Al		4	140	Ref [27]
G-134A	44	8	175	Ref [28]
NiCu-50/SBA-15		4	160	Ref [29]
Ru/C		2.5	165	Ref [S30]
Pt/C and Pt/Al_2O_3		8	160	Ref[S31]

References

S1 K. Umemoto, H. Takeuchi, H. Nakamura, A. Usuki, M. Fujita, *U.S. Pat. Appl. Publ.* 2008-237795, Sep 25, **2008**.

S2 G. Férey, C. Mellot-Draznieks, C. Serre, F. Millange, J. Dutour, S. Surblé, I. Margiolaki, *Science* **2005**, *309*, 2040.

S3 F. Liguori, P. Barbaroa, H. Sawa, *Appl Catal. A-Gen.* **2014**, *488*, 58.

S4 K. Nagaveni, A. Gayen, G. Subbanna, M. Hegde, *J. Mater. Chem.* **2002**, *12*, 3147.

S5 S. Samanta, A. K. Nandi, *J. Phys. Chem. C* **2009**, *113*, 4721.

S6 F. Harraz, S. El-Hout, H. Killa, I. Ibrahim, *J. Catal.* **2012**, *286*, 184.

S7 L. Chen, H. Chen, R. Luque, Y. Li, *Chem. Sci.* **2014**, *5*, 3708.

S8 P. Wang, F. Zhang, Y. Long, M. Xie, R. Li, J. Ma, *Catal. Sci. Technol.* **2013**, *3*, 1618.

S9 J. Niu, X. Huo, F. Zhang, H. Wang, P. Zhao, W. Hu, J. Ma, R. Li, *ChemCatChem* **2013**, *5*, 349.

S10 H. Wu, L. Zhuo, Q. He, X. Liao, B. Shi, *Appl. Catal. A-Gen* **2009**, *366*, 44.

S11 Y. Lang, Q. Wang, J. Xing, B. Zhang, H. Liu, *AIChE J.* **2008**, *54*, 2303.

S12 A. J. Amali, R. K. Rana, *Green Chem.* **2009**, *11*, 1781.

S13 S. Gao, M. Cao, W. Li, R. Cao, *J. Mater. Chem. A* **2014**, *2*, 12185.

S14 F. Zhang, J. Jin, X. Zhong, S. Li, J. Niu, R. Li, J. Ma, *Green Chem.* **2011**, *13*, 1238.

S15 S. S. Lee, S. N. Riduan, N. Erathodiyil, J. Lim, J. L. Cheong, J. Cha, Y. Han, J. Y. Ying, *Chem.-Eur. J.* **2012**, *18*, 7394.

S16 N. Arai, N. Onodera, A. Dekita, J. Hori, T. Ohkuma, *Tetrahedron Lett.* **2015**, *56*, 3913.

S17 Y. Wang, Y. Deng, F. Shi, *J. Mol. Catal. A-Chem.* **2014**, *395*, 195.

S18 Z. Wei, D. Thushara, X. Li, Z. Zhang, Y. Liu, X. Lu, *Catal. Commun.* **2017**, *98*, 61.

S19 H. Liu, P. Wang, H. Yang, J. Niu, J. Ma, *New J. Chem.* **2015**, *39*, 4343.

S20 Y. Ma, H. Wang, G. Xu , X. Liu, Y. Zhang, Y. Fu, *Chinese Chem. Lett.* **2017**, *28*, 1153.

S21 Y. Wang, S. Sang, W. Zhu, L. Gao, G. Xiao, *Chem. Eng. J.* **2016**, *299*, 104.

S22 G. Zhang, M. Zhu, Q. Zhang, Y. Liu, H. He, Y. Cao, *Green Chem.* **2016**, *18*, 2155.

S23 M. Hronec, K. Fulajtárová, I. Vávra, T. Sotákš, E. Dobrocka, M. Micušík, *Appl. Catal. B-Environ.* **2016**, *181*, 210.

S24 R. Fang, L. Liu, R. Luque, Y. Li, *Green Chem.* **2015**, *17*, 4183.

S25 X. Li, J. Deng, J. Shi, T. Pan, C. Yu, H. Xu, Y. Fu, *Green Chem.* **2015**, *17*, 1038.

S26 J. Guo, G. Xu, Z. Han, Y. Zhang, Y. Fu, Q. Guo, *ACS Sustainable Chem. Eng.* **2014**, *2*, 2259.

S27 H. Zhu, M. Zhou, Z. Zeng, G. Xiao, R. Xiao, *Korean J. Chem. Eng.* **2014**, *31*, 593.

S28 M. Hronec, K. Fulajtárova, M. Micušik, *Appl. Catal A-Gen.* **2013**, *468*, 426.

S29 Y.Yang, Z. Du, Y. Huang, F. Lu, F. Wang, J. Gao, J. Xu, *Green Chem.* **2013**, *15*, 1932.

S30 V. V. Ordomsky, J. C. Schouten, J. Schaaf, T. A. Nijhuis, *Appl. Catal. A-Gen.* **2013**, *451*, 6.

S31 M. Hronec, K. Fulajtarová, T. Liptaj, *Appl. Catal. A-Gen.* **2012**, *437-438*, 104.
